# The Role of Sex Hormones and Gene Expression in Cellular Properties of the vHPC to BLA Circuit in Anxiety Expression

**DOI:** 10.51894/001c.143845

**Published:** 2025-09-30

**Authors:** Victoria Fex, Chiho Sugimoto, Laura Furletti, Andrew Eagle, AJ Robison

**Affiliations:** 1 College of Osteopathic Medicine Michigan State University; 2 College of Osteopathic Medicine Michigan State University

## 09

### INTRODUCTION

The lack of understanding of the neurobiology underlying anxiety is a key reason for the increase in rates of anxiety disorders nationally. Thus, I am investigating the role of the ventral hippocampus (vHPC) to the basolateral amygdala (BLA) circuit in anxiety-like behaviors using mice. DeltaFosB is an immediate early gene transcription factor, and our lab has shown its expression in the vHPC to BLA circuit is required for anxiety-like behaviors. A separate protein, calreticulin, is important for proper protein folding and calcium homeostasis in cells, and we have shown it is a gene target of DeltaFosB. Finally, sex-based differences in anxiety exist, and they may result from differences in sex hormone effects on vHPC cell function. Thus, we will investigate the roles of DeltaFosB, calreticulin, and androgen receptor (AR) in the function of the vHPC-BLA circuit.

### OBJECTIVES

I hypothesize anxiety behaviors will be correlated with the decreased excitability of the vHPC CA1 glutamatergic neurons projecting on to the neurons in the BLA, and that excitability of these neurons is dependent on the function of androgen receptors, DeltaFosB, and/or calreticulin.

### METHODS

Whole cell slice electrophysiology will be the primary method used to investigate neuronal properties of the circuit. I will also use a variety of genetically modified mice with a knockout of specific proteins (DeltaFosB, calreticulin, and androgen receptors) while also keeping in mind potential sex-based differences. I used stereotaxic surgeries to inject viral vectors driving a green fluorescent protein (GFP) reporter specifically in the circuit. On top of understanding the basic properties of this circuit, I will perform behavioral stressors to induce anxiety-like states in mice to further investigate the effects of circuit excitability on anxiety-like behaviors. To do this, I will use both a subchronic and chronic stress paradigm that exposes animals to stressors such as tail suspension and foot shock, for either six days or three weeks respectively.

### RESULTS

This study is largely prospective, though there will be some results by May.

### CONCLUSIONS

Understanding the molecular underpinnings of excitability of the vHPC to BLA circuit will provide more specific pharmacological targets for drug development in anxiety treatment.

